# Advancing methodology for scoping reviews: recommendations arising from a scoping literature review (SLR) to inform transformation of Children and Adolescent Mental Health Services

**DOI:** 10.1186/s12874-020-01127-3

**Published:** 2020-09-29

**Authors:** Joanna K. Anderson, Emma Howarth, Maris Vainre, Ayla Humphrey, Peter B. Jones, Tamsin J. Ford

**Affiliations:** Department of Psychiatry, University of Cambridge, 18 Trumpington Road, Cambridge, CB2 8AH UK

## Abstract

**Background:**

There is consensus that health services commissioning and clinical practice should be driven by scientific evidence. However, workload pressures, accessibility of peer reviewed publications and skills to find, appraise, and synthesise relevant evidence are often cited as barriers to uptake of research evidence by practitioners and commissioners alike. In recent years a growing requirement for rapid evidence synthesis to inform commissioning decisions about healthcare service delivery and provision of care contributed to an increasing popularity of scoping literature reviews (SLRs). Yet, comprehensive guidelines for conducting and reporting SLRs are still relatively scarce.

**Methods:**

The exemplar review used as a worked example aimed to provide a readily available, comprehensive, and user-friendly repository of research evidence for local commissioners to help them make evidence-informed decisions about redesigning East of England Children and Adolescent Mental Health Services. In conducting the review, we were broadly guided by Arksey and O’Malley’s framework, however some modifications were made at different stages to better reflect the largely pragmatic objective of this review. This paper compares the methodology used with existing methodological frameworks for scoping studies, to add to the existing knowledge base.

**Results:**

We proposed the following advancements to the existing SLR frameworks: (i) Assemble a research team with complementary skills and expertise; (ii); Draw on expertise of external partners, particularly practitioners, decision-makers and commissioners who will be translating findings into practice; (iii) Pre-register the review protocol. Keep a detailed record of all steps and decisions and consider how they would impact on generalisability and utility of review findings; (iv) Use systematic procedures for literature searchers, selection of studies, data extraction and analysis; (v) If feasible, appraise the quality of included evidence; (vi) Be transparent about limitations of findings.

**Conclusions:**

Despite some methodological limitations, scoping literature reviews are a useful method of rapidly synthesising a large body of evidence to inform commissioning and transformation of CAMHS. SLRs allow researchers to start with a broader questions, to explore the issue from different perspectives and perhaps find more comprehensive solutions that are not only effective, but also accounted for their feasibility and acceptability to key stakeholders.

## Background

Evidence-based policy requires evidence, yet the policy and research cycles operate on vastly different timescales. There is consensus that health services commissioning and clinical practice should be driven by scientific evidence, but decision-makers often feel that gathering evidence may slow down innovation and overwhelm frontline staff [1]. Workload pressures and accessibility of peer reviewed publications are often cited as barriers to uptake of research evidence by commissioners [1, 2]. In addition to time constraints and accessibility, practitioners may lack the skills to find, appraise and synthesise relevant evidence [3]. The role of evidence-briefing services and information professionals is to find evidence relevant to local context and needs, and present them to commissioners as concise, actionable points [4, 5]. However, it is crucial that evidence guiding services and care provision comes from reliable sources, is critically appraised, and interpreted in the light of the overall body of available evidence [6].

Traditionally, systematic reviews are considered the most robust and reliable evidence synthesis method to inform evidence-based healthcare, answer clinically meaningful questions about treatments [7, 8], and particularly guide the development of trustworthy clinical guidelines [9]. Systematic reviews generally include evidence from randomised controlled trials, and observational epidemiological studies, however, recently the importance of including qualitative evidence has become increasingly recognised [7, 10]. Systematic reviews, although thorough, are also very resource-intensive and may take a long time to complete [11]. A growing requirement for rapid evidence synthesis in response to the needs of decision-makers has increased the popularity of rapid literature reviews [11]. Rapid reviews are designed to expedite the review [12, 13] through a number of compromises including reducing the scope, reducing the number of sources searched (often by including only systematic reviews or economic evaluations), exclusion of grey literature and foregoing double screening and data extraction [11, 12]. Systematic and rapid reviews are usually very narrow in focus and aim to answer a small number of narrowly defined questions. In recent years Scoping Literature Reviews (SLRs) have become an increasingly popular method of knowledge synthesis. SLRs offer a rapid method of mapping key concepts in a research area and identifying the main sources and types of evidence available [14]. One of the aims of SLRs is to synthesise and disseminate research results to audiences that otherwise would not have time or resources to conduct a review themselves [15–17]. Peters et al. (2015) stress the utility of SLR in informing evidence-based practice through “( …) examination of a broader area to identify key gaps in the research knowledge base, clarify key concepts and report on types of evidence that address and inform practice in the field”. Table 1 below outlines the key differences between systematic, rapid and scoping literature review.

**Table 1:** A comparison of systematic, rapid and scoping literature review

Review type	Aims	Research question/ scope	Process	Literature searches	Inclusion criteria	Quality of evidence	Bias	Reviewers
**Systematic**	To inform clinical practice.	Narrow and well-defined.	Explicit, predefined, sequential process rigorously followed.	As exhaustive as possible; using pre-defined search strategy.	Predefined, (PICOS criteria)	Quality of evidence assessed and reported.	Systematic, explicit methods to minimize bias.	Requires at least two reviewers for study selection, data extraction and quality appraisal.
**Rapid**	To inform service provision; caution when informing clinical practice.	Narrow and well-defined.	‘Trimmed down’ systematic review process; shortcuts to minimise resources used.	Reduced list of sources searched; using search tools that facilitate finding literature.	Predefined, (PICOS criteria).	Quality of evidence assessed and reported.	Shortcuts may introduce bias.	Does not require two reviewers for study selection, data extraction and quality appraisal.
**Scoping**	To map evidence, identify knowledge gaps, inform policy and practice.	Broader, initially parameters may not be clearly defined (e.g. type of intervention).	Iterative process, no formal requirement to include all steps.	Focus on comprehensiveness and breadth when defining search terms and sources. Can be altered at later stages.	Often developed post-hoc as reviewers become more familiar with available evidence.	No requirement to assess the quality of evidence.	Omitting/ altering steps may introduce bias.	Required number of reviewers not specified.

Despite growing popularity of SLRs [18] our searches identified only three complete guidelines for conducting and reporting these studies [15–17]. The first comprehensive SLR framework was published by Arksey and O’Malley (2005) [15] (see Table 2), and has since been advanced [16, 17, 19–21] (see Table 3). However, only two enahnced frameworks proposed by Levac et al. [17] and The Joanna Briggs Institute [16] outline a step-by step process of conducting SLRs. Other advancements comment and expand on particular stages of the process [19–21, 24] without offering a comprehensive guide. As a result researchers, particularly those not having much experience of reviewing evidence, have to collate existing guidelines and decide which modifications to include. This is likely to result in methodological inconsistencies across scoping studies, and varied quality of outputs. This paper presents a comprehensive guideline for conducting SLRs based on previous frameworks and its’ advancements, as well as our team’s additions.

**Table 2:** Arksey and O’Malley SLR framework

Authors	Aims	Steps
Arksey, O’Malley (2005) [15]	▪ To map the extent, range and nature of research activities undertaken in a field of interest. ▪ To establish the need for, and a potential cost of conducting a full systematic review. ▪ To identify research gaps. ▪ To synthesis and disseminate research results.	(1) Identify research questions. The authors recommend to maintain a wide approach and initially avoid defining parameters clearly (e.g. type of intervention, population etc.) to ensure of coverage. (2) Identify relevant studies. It is recommended that researchers focus on comprehensiveness and breadth when making a decision about which search term to use, and what sources of evidence to search. (3) Studies selection. Unlike in systematic review, in SLR inclusion and exclusion criteria are not predefined but developed post hoc as researchers familiarise themselves with available evidence. (4) Chart the data. The authors recommend using ‘narrative review’ or ‘descriptive analytical method’ to sort evidence according to key issues and themes that are of particular interest, as defined by research questions and purpose of the SLR. (5) Collate, summarise and report the results. The authors suggest applying analytic framework or thematic construction to present an overview of available evidence (numerical analysis), but argue that SLR, unlike systematic review, is not meant to aggregate and synthesis findings.

**Table 3:** SLR frameworks advancing Arksey’s and O’Malley’s methodology

Authors	Aims	Proposed advancements
Anderson et al. (2008) [22]	*“Scoping studies are concerned with contextualizing knowledge in terms of identifying the current state of understanding; identifying the sorts of things we know and do not know”.*	*None*
Levac et al. (2010) [17]	*Same as outlined by Arksey and O’Malley (2005)*	(1) Clarify research questions by linking them with purpose and rationale for conducting the SLR (2) Balance feasibility with extensiveness of the review process, ideally through consultations within a research team representing relevant content and methodological expertise (3) Use an interactive team approach to data selection and extraction (4) Provide quantitative summary and qualitative thematic analysis in the report, as well as discussing implications for research and practice (5) Include consultations with stakeholders as mandatory knowledge translation component of scoping review
Daudt et al. (2013) [20]	“S*coping studies aim to map the literature on a particular topic or research area and provide an opportunity to identify key concepts; gaps in the research; and types and sources of evidence to inform practice, policymaking, and research”*.	(1) Assess quality of included study (2) Trialing data charting method to ensure consistency
The Joanna Briggs Institute (2015) [16]	*“The value of scoping reviews to evidence-based practice is the examination of a broader area to identify gaps in the research knowledge base, clarify key concepts, and report on the types of evidence that address and inform practice in the field. Scoping reviews also may be carried out to determine not only the extent of the research available regarding a topic, but also the way the research has been conducted”.*	(1) Develop an a-priori protocol that clearly defines objectives and research questions, which in turn determine inclusion/exclusion criteria defined using Population, Concept and Context (PCC). (2) Clearly articulate the core concept examined by the SLR to guide the scope and breadth of evidence covered, and determine the outcomes.
Peters et al. (2015) [23]	*“Scoping reviews have great utility for synthesizing research evidence and are often used to map existing literature in a given field in terms of its nature, features, and volume. (…) they may also be undertaken as exercises in and of themselves to summarize and disseminate research findings, to identify research gaps, and to make recommendations for future research”.*	*None*
Colquhoun et al. (2014) [19]	*“A scoping review or scoping study is a form of knowledge synthesis that addresses an exploratory research question aimed at mapping key concepts, types of evidence, and gaps in research related to a defined area or field by systematically searching, selecting, and synthesizing existing knowledge”.*	To improve the quality, transparency and completeness of reporting, and enable critical appraisal, and increase transparency the authors recommend applying The Enhancing the QUAlity and Transparency Of health Research (EQUATOR) reporting guidelines.
Kahalil et al. (2016) [21]	*“Scoping reviews are used to assess the extent of a body of literature on a particular topic, and often to ensure that further research in that area is a beneficial addition to world knowledge”.*	(1) Clarify and link the purpose of the review with research questions. (2) Use a three-step literature search to balance feasibility and comprehensiveness (3) Study selection by the team (4) Present data in both tabular and narrative formats (5) Identify implications to policy, practice and research
Tricco et al. (2018) [24]	*“(SLRs) may examine the extent (that is, size), range (variety), and nature (characteristics) of the evidence on a topic or question; determine the value of undertaking a systematic review; summarize findings from a body of knowledge that is heterogeneous in methods or discipline; or identify gaps in the literature to aid the planning and commissioning of future research”.*	To improve the quality of reporting the authors recommend using PRISMA-ScR (Preferred Reporting Items for Systematic reviews and Meta-Analyses extension for Scoping Reviews) checklist. It provides a comprehensive guide on how to systematically and exhaustively report scoping studies.
Our team’s contribution	*A scoping review is a method of knowledge synthesis that can facilitate synthesis and summary of a large body of evidence in a relatively short time. One of its applications is to disseminate research results and highlight their practical implications to guide and support decision makers, who do not have time, skills or resources to synthesis and critically appraise evidence themselves,*.	(1)Assemble a research team with complementary skills and expertise. (2) Draw on expertise of external partners, particularly practitioners, decision-makers and commissioners who will be translating findings into practice. (3) Pre-register the review protocol. Keep a detailed record of all steps and decisions. Note rationale for each decision and consider how it would impact on generalisability and utility of review findings. (4) Use systematic procedures for literature searchers, selection of studies, data extraction and analysis. (5) If feasible, appraise the quality of included evidence. (6) Be transparent about limitations of findings.

First, we describe and discuss the methodology used to conduct an SLR. To this end we provide an exemplar SLR and describe how the specific purpose of the exemplar SLR defined and shaped its’ subsequent stages. We describe the steps taken from defining the research questions, charting the evidence, and data analysis to the final presentation of the findings. We highlight additional steps we have taken and describe how our methodology deviated from the original Arksey’s and O’Malley’s framework [15] to aid the pragmatic purpose of our SLR. This paper focuses only on methodological aspects of the SLR; findings from the review are published elsewhere [25–27].

## Method

### The exemplar

The SLR used as a worked example in this paper aimed to inform the redesign of East of England Children and Adolescent Mental Health Services (CAMHS) to create more accessible, user-friendly and efficient service for young people and their families. It was conducted as part of a multifaceted research initiative that was undertaken to support local operationalisation of the recommendations outlined in “Future in Mind” – a taskforce report outlining the improvements needed in the way CAMHS are organised, commissioned and provided, to optimise children’s and young people’s mental wellbeing in the UK [28]. The pragmatic objective was to examine how, considering the local context, the services offered in the region could be redesigned to best support children and young people who require mental health provision at different treatment intensity levels. The outputs from this SLR informed the development of the Local Transformation Plan [29] that guided the subsequent local CAMHS redesign.

In conducting the review, we were broadly guided by Arksey and O’Malley’s framework [15], however some modifications were made at different stages to better reflect the largely pragmatic objective of this review. These included:

1. Predefining research questions, breadth and coverage, and inclusion/exclusion criteria to reflect the key priorities for local CAMHS redesign selected by stakeholders. Rather than being informed by research objectives [15], this process was guided by the outcomes of consultations with stakeholders, and our understanding of the local context as described by expert practitioners.
2. Keeping a detailed record of every decision made about breadth and coverage of the SLR, inclusion/exclusion criteria and data analysis, noting the rational for it, and considering how it impacts on generalisability and practical applications of SLR findings.
3. Consultations with a broad range of experts at every stage of the review. We primarily relied on guidance from practitioners, commissioners and other decision-makers to ensure that the review is ‘fit for purpose’ i.e. to inform decisions about redesigning existing services and commissioning new ones, and to provide a practical guide on how to implement and evaluate them.
4. We applied rigorous methods used for systematic reviews to maximise robustness and ensure replicability of our methodology. These include using predefined search strategy and inclusion/exclusion criteria, and having two reviewers preform study selection and data extraction.
5. Although we did not undertake it in the exemplar review, we strongly recommend appraising included studies for quality.
6. Include a detailed description of limitations of findings and of their practical applications.

## Results

### SLR stage 1: defining research questions, coverage and breadth, and inclusion/exclusion criteria

#### Defining aims and research questions

Aims and research questions were formulated to reflect the pragmatic aim of the SLR i.e. guiding the operationalisation of key priorities for local CAMHS transformation. These priorities were identified through Delphi-method consultations [30] with stakeholders including children and young people (CYP), parents/carers, clinicians/other professionals working with CYP, and local healthcare commissioners [30]. The SLR aimed to: (i) provide a readily available repository of evidence for effectiveness, acceptability and feasibility of implementing the service improvements identified by stakeholders as key priorities; (ii) enable commissioners to make evidence-informed decisions about redesigning currently provided services and implementing new ones; (iii) guide change processes; (iv) measure outcomes. These objectives led to the following, broad research questions:

1. What are the effective ways of delivering services that were prioritised for CAMHS transformation?
2. What ways of delivering services that were prioritised for CAMHS transformation are feasible and acceptable for CAMHS users?
3. What are the effective procedures for implementing services that were prioritised for CAMHS transformation?
4. What are the effective ways of measuring transformation outcomes?

#### Defining coverage, breadth and inclusion/exclusion criteria

**Fig. 1:**
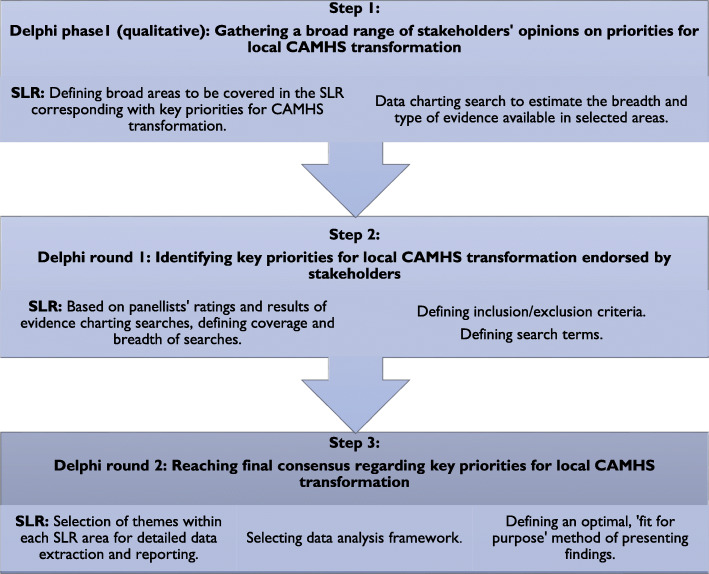
Process of defining coverage of the SLR

The pragmatic purpose of the SLR determined coverage corresponding with key priorities for local CAMHS transformation identified through the three-stage Delphi study [30]. The process is outlined in Fig. 1 below.

**Delphi phase 1 (qualitative)**: Following completion of the first round of the Delphi, JKA conducted an “evidence charting” search to understand the amount and type of evidence available for each key priority identified. A systematic search strategy was then developed to reflect key priorities identified through the first round of the Delphi (see Table 4).

**Table 4:** Coverage and breath of the SLR based of the areas identified through the Delphi study

***Areas of service provision***	***Key priorities identified - Delphi phase 1 (qualitative)***	***Areas identified through evidence charting search***	***Final list key priorities to be covered in the SLR – Delphi round 1***
***Prevention and promotion of MH and wellbeing***	Role of family, community and schools (prevention and promotion)	Community based prevention School based prevention Suicide prevention Substance abuse prevention	School based prevention Suicide prevention
	Communication about mental health Education about mental health Information about available CAMHS Promotion of CAMHS	Education and rising awareness	Education and raising awareness
	Developing professional skills/staff training	School staff training	
	Using internet and mobile technologies	Web-based interventions	Technology enabled MH interventions
***Screening and identification***	Screening/early detection Initial assessment/ early intervention	Early intervention Screening tools	Screening tools
	Role of family, community and schools (identification)	School based screening Screening in healthcare settings	School based identification Screening in healthcare settings
	Using mobile technologies		Technology enabled identification
***Access to CAMHS***	Access: open vs. referral based Access to CAMHS (specialist care) Referrals system	Access/referrals (general) Pathways Barriers for access/referral	Barriers for access and referral
	Access times	Waiting times	Waiting times
	Accessible settings	Improving access	Improving access
		User experience	User experience
***Provision of CAMHS***	Provision of evidenced based practice Provision of individually tailored services / personalization of CAMHS Role of family, community and schools in CAMHS provision Role of healthcare professionals (other than mental health) in CAMHS provision	Service delivery models Advocacy Therapeutic alliance	Service delivery models
	Continuity of care Multidisciplinary CAMHS Holistic approach	Integrated/comprehensive services	Integrated/comprehensive services
		Interventions delivered using mobile technologies	Technology enabled MH interventions
***Service evaluation and improvement***	None	Quality indicators/service evaluation	Quality indicators
		Quality improvement initiatives Redesign/implementation	Service redesign and implementation
		User experience/satisfaction	User experience/satisfaction
	Outcome monitoring		Outcome monitoring

Relevant Medical Subject Headings (MeSH) terms and key words in titles and abstracts were searched (see Additional file 1, Table 1). Search results for each key priority were sorted according to relevance (using EBSCO Host results sorting tool) and the first 2000 most relevant abstracts were retrieved and screened using preliminary inclusion/exclusion criteria [15] (See Additional file 1, Table 2). Full texts of included abstracts were retrieved and screened, and full text publications that met inclusion criteria were assigned to categories corresponding with key priorities for CAMHS transformation identified through the first round of the Delphi (See Additional file 1, Table 3).

**Delphi round 1:** Outcomes of the evidence charting searches, together with findings from the second round of the Delphi study guided the research team’s discussion about the breadth and depth of the definitive literature searches, inclusion of any additional areas, and defining the final inclusion/exclusion criteria. The research team compared Delphi statements for which the highest consensus was achieved in regard to their particular importance for local CAMHS transformation, against areas identified through evidence charting searches to define the coverage and depth of definitive literature search. Additionally, the team consulted external experts about the completeness of the list of key priorities for CAMHS transformation and areas important for designing and delivering effective CAMHS that were not featured in the Delphi results, but in their opinion should be included. (See Table 4).

**Delphi round 2:** Following the third and final round of the Delphi, the research team selected the themes within each prioritised area for data extraction and reporting (See Table 5).

**Table 5:** Themes identified within each key priority area

***Areas of service provision***	***Key priorities for local CAMHS redesign***	***Themes identified***
***Prevention and promotion of MH and wellbeing***	School based prevention	Evaluation of school-based prevention programmes Development of school-based prevention programmes
	Suicide prevention	Evaluation of school-based suicide prevention programs Evaluation of community-based suicide prevention programs Development and description of suicide prevention programs
	Education and raising awareness	Evaluation of school MH education/awareness/anti-stigma programs Assessment of MH literacy Assessment of attitudes towards MH problems
***Screening and identification***	School based identification	Development of school-based identification programmes Evaluation of school-based mental health identification programmes
	Screening in healthcare settings	Outcomes of MH screening in healthcare settings MH screening in healthcare setting and subsequent referral/use of MH services Parental attitudes towards MH screening in healthcare settings MH professionals’ attitudes towards MH screening in healthcare settings
	Screening tools	Development and psychometric properties of screening measures Feasibility/ acceptability/ utility of screening measures
***Access to CAMHS***	Barriers for access and referral	Organizational and administrative barriers for access to CAMHS Users’ and healthcare professionals’ perspectives on barriers to seeking help/ access to CAMHS/ treatment engagement Demographic and socioeconomic factors associated with seeking help/ access to CAMHS/ treatment engagement
	Wait times and improving access	Interventions to reduce wait times and/ or improve access to CAMHS Improving access through providing MH services in schools/primary care settings Impact of wait times on attendance/ treatment engagement Service/ patient factors associated with wait times
***Provision of CAMHS***	Service delivery models	Interagency collaboration Coordination of care School-based MH services
	Integrated/comprehensive services	Evaluation of an integrated care model
	Technology enabled MH interventions	Evaluation of technology enabled MH interventions Attitudes towards technology enabled MH interventions Development and description of technology enabled MH interventions
***Service evaluation and improvement***	Service redesign and implementation	Implementation of services Diffusion of innovations Service improvement/redesign
	User experience/satisfaction	Service users’ experience of CAMHS Service users’ satisfaction with CAMHS Development and psychometric properties of users satisfaction with services measures
	Outcome monitoring	Routine outcome monitoring Service outcomes Development and psychometric properties of outcome measures
	Quality indicators	Development and psychometric properties of quality measures Quality assessment Development of quality standards

#### Defining the final inclusion/exclusion criteria

The methodological framework for conducting scoping studies recommends that initial inclusion/exclusion criteria are developed at the onset of the study, however these are reviewed and if necessary revised post hoc, in light of search results and researchers’ increased familiarity with evidence [15, 17]. We predefined inclusion/exclusion criteria to exclude papers that were not empirical, not reviews of other studies or not policy documents or guidelines (e.g. commentaries, letters, book reviews). To further define inclusion/exclusion criteria we applied Population, Concept and Context criteria (PCC) as suggested by Joanna Briggs Institute guidelines [16]. Once again, our inclusion/exclusion criteria reflected the primarily pragmatic purpose of the review. For example, we only include studies conducted in developed countries where the contexts and settings are likely to be similar to the UK. We decided to exclude studies that did not report CYP mental health (MH) or wellbeing outcomes, and to include studies in which the intervention was delivered to adults, as long as the aim was to influence CYP’s MH outcomes. Due to time restrictions and the large number of retrieved publications, we excluded studies focusing on emotional or behavioural symptoms associated with non-mental health disorders (e.g. autism spectrum disorders) and studies focusing solely on single therapeutic approaches (e.g. cognitive behavioural therapy (CBT) for depression). We decided that the focus must include service delivery and be relevant to community care, regardless of persons or organisation delivering the intervention, and severity or duration of mental health condition, given the purpose of the review and the large number of published reviews of the effectiveness of different therapeutic approach in addressing various MH problems (e.g. CBT for depression). We included studies of any design. See Table 6.

**Table 6:** Scoping literature review inclusion/exclusion criteria

	EXCLUDE IF:
1	Not written in English.
2	Published before January 1990.
3	Not empirical, not evidence based, not reviews of other studies or not a policy document/guideline (exclude commentaries, letters, book reviews).
4	Not directly or indirectly focused on mental health service users age 0–25 years (i.e. studies with parents/carers of mental health service users, service providers will be included).
5	No focus on mental health or mental disorders. Exclude if symptoms are associated with non-mental health disorder (e.g. behavioural problems associated with ADS)
6	Does not report children, adolescents or young people’s mental health or wellbeing outcomes, if intervention or programme targets adults’ mental health.
7	No focus beyond treatment type. The focus must include service delivery and be relevant to community care (regardless of persons or organization providing services, and severity or duration of mental health condition).
8	Services are not delivered in community settings (e.g. primary care, schools, youth centres).
9	Describes children and adolescents mental health services in developing countries (according to World Economic Situation and Prospects 2014).

### SLR stage 2: identifying relevant studies

To develop search terms for definitive database searches JKA re-read papers included after screening publications identified through evidence charting searches (see Additional file 1, Table 2), and listed key words and index terms used in each priority area. This list guided the development of terms for electronic bibliographic databases searches (see Additional file 1, Table 4). Search strategies for all areas were discussed with a subject librarian and agreed by the research team, and each strategy was trialled to see if it produces accurate results. Databases and other sources searched are listed elsewhere [26]. To ensure that the search strategy was replicable, each search was stored exactly as run, together with search set numbers, and the number of records retrieved. For each search we separately recorded search date and the period searched, and any language or publication status restrictions.

### SLR stage 3: study selection

As recommended for systematic reviews, prior to commencing the selection process initial inclusion/exclusion criteria were pilot tested by JKA on a sample of 20 papers from each identified area. Results of pilot screening were recorded in a table including reference, key priority area, theme, inclusion/exclusion decision, reason for exclusion (if excluded), and comments on paper’s relevance if there were doubts whether it should be included or excluded. Results of pilot screening were discussed within the research team with particular focus on applying refined inclusion/exclusion criteria (Table 5) and relevance. The studies’ selection process comprised the following stages:

1. All search results from both electronic database searches and hand searches were merged using the reference management software (EndNote). Duplicate records of the same report were removed. Documents retrieved in grey literature searches were filed separately in Excel database and separately screened for relevance.
2. Titles and abstracts of all papers and grey literature documents were examined to remove obviously irrelevant papers (e.g. not in English, not concerning MH services).
3. Abstracts/executive summaries of remaining papers were screened against inclusion/exclusion criteria.
4. Full texts of remaining, potentially relevant papers were retrieved and multiple reports of the same study were linked.
5. Full texts were examined for compliance with inclusion/exclusion criteria. After completing abstracts and full text screening another member of the team double screened 10% of papers in each review area. Team discussions allowed disagreements and boundary papers to be included/excluded via consensus. We kept a detailed record of the outcomes of each stage, including rejected papers and reasons for rejection. Additionally, each screening researcher identified and recorded main themes for each paper in the key priority area. (See Table 5).

### SLR stage 4: data extraction

After completing the last round of the Delphi study, we collated a final list of key priorities for local CAMHS redesign [30]. This list informed the final selection of themes within each identified area for detailed extraction and reporting (see Table 5).

Before data extraction the lead researcher developed an extraction table to be trialled by members of the research team. Because the review included a variety of evidence types i.e. papers describing qualitative and quantitative studies, process evaluation studies, theory and framework papers, discussion and opinion papers, policy documents, we developed different forms to extract different type of evidence. This aimed to maximise the amount of meaningful information extracted, and to facilitate further narrative synthesis process. Data for each key priority area identified through the Delphi study were organised in a separate table(s). Extracted data were organised under the key themes identified in the previous stage (See Table 5). Identifying key themes was an iterative process with some themes added, deleted, or replaced by a new, more relevant ones as the analysis progressed.

### SLR stage 5: collating, summarizing and reporting results

The pragmatic purpose of SLR guided the selection of frameworks to summarise and report results. We needed a ‘fit for purpose’ method of analysing and synthesising findings that would be both robust, systematic and scientifically driven, yet presented in a way that is useful and accessible for commissioners and other decision-makers.

Firstly, we provided a numerical account of findings for each key priority area identified. We reported years of publication, countries where studies were conducted and study designs or type of publications in both numerical and graphical form. This was to enable readers to establish whether evidence sources were up to date and relevant in UK context; for example, in some areas only a small fraction of reported studies were conducted in the UK, or most studies were dated before the year 2000. Although we were not assessing the quality of evidence, we noted that in some areas majority of publications were opinion and discussion papers, rather than primary research or systematic reviews. It was important to highlight, as any conclusions or recommendations made in regard to these priority areas reflected experts’ opinions, rather than research evidence.

Before commencing narrative data analysis, we created a logic map to represent all themes in each key priority area, to illustrate how some themes from the same or different areas overlap or complement each other, and to help us organise the synthesis to provide the most comprehensive picture (see Additional file 2). To summarise available evidence, draw conclusions, make clear recommendations regarding most appropriate, feasible and acceptable ways of delivering services, effective procedures for implementing these services and measuring outcomes, we carried out a narrative synthesis of evidence for each key priority for local service transformation. The narrative synthesis process was broadly guided by the framework proposed by Popay et al. [31]. This framework, applicable both to effectiveness and implementation reviews, comprises four main phases that are iterative rather than linear. The framework was developed mainly to synthesis data gathered through systematic literature review, thus we adjusted it to synthesis broader and more varied evidence.

Phase I: Developing theory of change. Explaining a theoretical model of how, why and for whom an intervention works is a recommended rather that mandatory stage of narrative synthesis [31]. When conducting a systematic review, a theory of change is usually developed in its initial stages to understand the theory behind the intervention and to inform the decision about review questions, and what type of studies to include. The theory of change can guide an interpretation of findings an assessment of its applicability. However, in systematic reviews, review questions are well defined and usually narrow, regarding only interventions with particular focus or design. In contrast, the described SLR had a much broader objective with research questions that reflected different stages of service transformation including development of new services or redesigning existing ones, service implementation and measuring transformation outcomes. The review focused on a number of areas of service delivery identified as key priorities for local CAMHS transformation. In some priority areas (mainly regarding service delivery model, service redesign and implementation), a majority of publications were discussion and opinion papers and case studies, while in other areas (e.g. technology delivered MH interventions, education and rising awareness) we identified a large number of empirical evaluations of the effectiveness of interventions targeting various outcomes. For the latter, we attempted to outline a theory of change post hoc to describe mechanisms and factors that determined intervention effectiveness in achieving intended outcomes, and contributed to its successful or failed implementation.

**Fig. 2:**
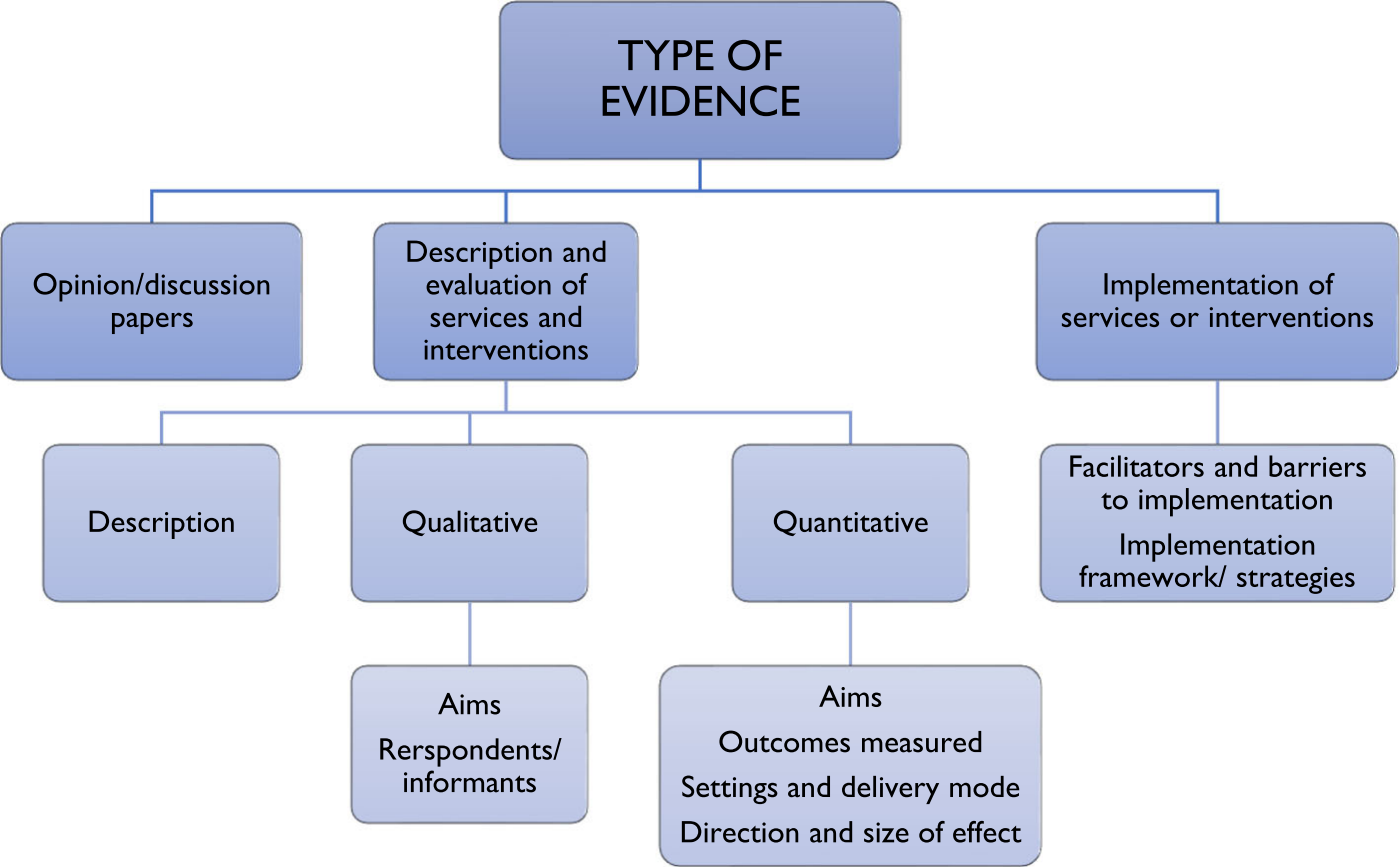
Grouping of papers for preliminary data synthesises

Phase II: Developing a preliminary synthesis of evidence. Evidence synthesis was conducted separately for each key priority area, however we identified some overlaps between themes in different areas (see Fig. 2). Where themes overlapped, data from different areas were merged to provide a comprehensive summary. To develop a preliminary synthesis of evidence for each priority area we organised evidence on a number of levels, to identify patterns in factors that contributed to the effectiveness of services or interventions. Initially, we grouped papers in each area based on key themes we assigned them to (see Table 5). In some instances it was sufficient to carry out preliminary data synthesis (e.g. development and psychometric properties of outcome measures). If further organising of evidence was required, we grouped the papers as follows, adjusting the grouping (depending on the nature of evidence available in each area reviewed):

From the analysis of information included in the extraction tables, we identified some initial, broad themes and patterns to explore further in the next stage. For each priority area we also considered potential limitations resulting from both the nature of available evidence (e.g. lack of UK studies) and review process (e.g. decisions about search strategy), to consider when translating findings from the review into practice.

Phase III: Exploring relationships in data. The aim of this is to identify factors explaining differences in direction and size of effects across included studies, and factors that might explain differences in facilitators and barriers to successful implementation. The SLR aimed to provide an evidence-base to guide the transformation of local CAMHS in each identified priority area. In practice we were aiming to develop a robust, credible, yet concise and user-friendly summary of evidence (See Additional file 3) readily available for commissioners to support them in making decisions about developing, implementing and evaluating services most appropriate in different contexts and for different user groups. We explored relationships between data in each key priority area to determine which ways of delivering services are the most feasible, clinically and cost-effective, and acceptable for users in different contexts, and to identify common factors contributing to effectiveness and acceptability. Additionally, we described factors that may serve as barriers and facilitators for effective implementation. Finally, we explored optimal ways of measuring potential transformation outcomes that would provide rich, reliable data, while minimizing burden on service users and clinicians.

Phase IV: Assessing the robustness and strength of evidence for drawing and generalising conclusions. SLR guidelines explicitly state that scoping studies do not aim to assess the quality of available evidence, but only report extent, range and nature of research activities undertaken in a field of interest [13, 16]. We accordingly provided a narrative description of types of available evidence, identified gaps for each key priority area, and elucidated what impact this may have on the robustness of any recommendations made and the translating findings into practice (See Additional file 3). We have not assessed the quality of evidence since our SLR included grey literature. However, where feasible, we would recommend assessing the quality of included studies to increase the reliability of findings and recommendations for practice.

## Discussion

Only in recent years scoping literature reviews have become an increasingly popular method of evidence synthesis, particularly in relation to healthcare service delivery and provision of care [22]. Earlier SLR frameworks described evidence mapping, identifying key concepts and research gaps in the field as primary objectives of scoping studies [14, 16, 19, 20, 24]. However, more recently, authors have placed more emphasis on conducting SLRs to inform evidence-based provision of services and disseminate findings to audiences who could benefit from access to research evidence, but lack capacity or skills necessary to collate them themselves as [7, 19, 20, 23, 32]. Scoping reviews generally aim to answer broader questions than systematic and rapid reviews, and leave room for iterations introduced during the review process. This may be an advantage when answering questions about provision of services, as healthcare systems are dynamic and often have to rapidly respond to changing circumstances. Most recent advancements in SLRs’ methodology have introduced more rigour to the review process [19, 24], increasing replicability, reliability of findings, and recommendations for practice. These advancements bridge the gap between SLRs and systematic and rapid reviews, i.e. allowing to rapidly review a large body of diverse evidence without sacrificing methodological rigour or scope.

The main aim of the exemplar SLR was to develop an evidence-base to inform and guide transformation of local CAMHS. Our objective was to provide a readily available, comprehensive, and user-friendly repository of research evidence for local commissioners to help them make evidence-informed decisions about redesigning currently available CAMHS services and commissioning new ones. Our decision to conduct a scoping rather than a systematic or rapid literature review was dictated by a number of factors. Firstly, we have started with a broad scope that we then gradually narrowed down based on the outcomes of the Delphi study. Secondly, our focus was not on the effectiveness of certain therapeutic approaches or interventions delivered in CAMHS. If that was the objective, a systematic review would have been far more appropriate [9]. Yet, our focus was on effective, feasible, and acceptable ways of delivering services prioritised by stakeholders, as well as on implementation strategies that would facilitate new service’s adoption and spread, and on measuring outcomes. Our initial charting searches showed that if we only included peer-reviewed publications (as recommended for a systematic review), we would not have been able to present a full picture and make comprehensive recommendations since a large body of relevant evidence were published in grey literature (i.e. policy documents, government bodies’, third sector organisations’ and think-tanks’ reports). Moreover, the majority of these documents did not adhere to research reporting guidelines, and would not pass the quality appraisal. Nevertheless, they offered valuable information that could support commissioners in making decisions about redesigning existing services and commissioning new ones. Finally, unlike in systematic reviews, our questions and methods were driven by practice rather than research objectives. Although we identified knowledge gaps, our primary focus was to collate evidence to support CAMHS transformation, i.e. evidence that could be rapidly translated into practice. We only reported on areas that were relevant from the practice point of view and the manner of reporting was driven by practical requirements, thus the review was unlikely to be exhaustive.

Our methodology was broadly guided by Arksey and O’Malley’s framework [15], however, some modifications were made at different stages to better reflect the largely pragmatic objective of this review. We needed a ‘fit for purpose’ method of analysing and synthesising findings that would be both robust, systematic and scientifically driven, yet presented a way that is useful and accessible for commissioners and other decision-makers.

The most complex aspect of the review was to define its coverage and breadth. Unlike in most reviews, it was an iterative process primarily driven by practical requirements, rather than research purpose. It was informed by outcomes of each round of the Delphi consultations to determine key priorities for local CAMHS redesign. We have relied not only on the expertise of the research team members, but also drawn on the expertise of other mental health researchers, CAMHS clinicians, services managers, commissioners and other decision-makers. We kept a very detailed record of the process, noting all sources of information and factors that guided our decisions about the review’s coverage and breadth.

Inclusion and exclusion criteria were also defined with practical purpose in mind; however, it is important to note that decisions about which studies to include created some limitations to generalisability and utility of our findings. For example, we decided to only include studies conducted in developed countries. The rationale for this decision was that we needed to focus on contexts and settings similar to the UK ones, as the findings were meant to inform local practice. We assumed that service implementation and sustainability in developing countries is likely to be influenced by different factors that may not be so prominent in the UK (i.e. very limited resources, remote geographical locations etc.).

We applied rigorous processes used in systematic reviews to define search strategy, select papers for inclusion, extract data and analyse data. We kept detailed records documenting every step to ensure our methods are systematic, verifiable and replicable. Initially, we extracted and data and synthesised evidence separately for each key priority area. Subsequently, we explored relationships between data in each area to determine which ways of delivering services were the most feasible, clinically and cost-effective, and acceptable for users in different contexts. We also identified common factors contributing to effectiveness, feasibility and acceptability. Finding these commonalities allowed us to synthesise a very large amount of data relatively quickly an in a succinct manner. Our outputs were concise summaries of findings and recommendations, focusing primarily on practical implications, foregoing methodological considerations (see Additional file 3). We did not conduct a critical appraisal of evidence, since we included grey literature. We noted limitations to our findings which mainly resulted from the lack of UK studies, lack of empirical evidence in some areas (i.e. retrieved publication were predominantly discussion and opinion papers), as well as methodological decisions we made.

### Limitations of proposed methodology

It is important to highlight that described methodology has a number of limitations. A SLR informed by practical requirements is unlikely to be exhaustive. Research driven SLR are usually comprehensive and cover a distinct topic, while practical questions are likely to be focused on a narrow area and not necessarily considering broader context. Practical purpose will also dictate the coverage and breadth of the review, inclusion/exclusion criteria, and to some extent the data analysis methods. In most cases this would mean focusing on evidence relevant to a specific, usually narrow context resulting in significant constraints with regard to the generalisability and applications of the findings. Focusing on a narrow, specific context restricts conclusions about knowledge gaps thus caution is needed when making conclusions about the need for conducting a full systematic review, or recommending direction for future research.

SLR aiming to inform practice often includes varied types of evidence, some of them not meeting the quality standards for research and reporting. While findings from those reviews can inform broadly defined service provision, implementation or redesign, they are not a trustworthy evidence-base to determine an effectiveness of a particular service or intervention; in other words they are not design to support decisions about using specific interventions or treatments. These decisions are more reliably guided by findings from systematic reviews.

Although conducting a SLR is definitely quicker than a full systematic review, it is still quite time- and resource-intensive process. Particularly with SLRs aiming to inform practice, the initial stages (i.e. the process of defining research questions, coverage and inclusion criteria) can be quite lengthy, since they require a coordinated input from different groups of experts and stakeholders. If resources are an issue, it might be more feasible to conduct a rapid review, however, considering literature searches restrictions and stringent inclusion/exclusion criteria, the findings are likely to only partially reflect available evidence.

Finally, although involving commissioners and decision-makers in defining research questions and scope of a review is very beneficial and useful when it comes to informing practice, there are also downsides that need to be considered. Different decision-makers may have different, often competing priorities that do not always align with those of other stakeholders (e.g. service users and clinicians). Designing a review that addresses all different priorities may be very challenging, and foregoing some to give precedence to others may result in limited practical applications and usefulness of findings.

## Conclusions

Despite some methodological limitations, SLRs are a useful method of rapidly synthesising a large body of evidence to inform commissioning and transformation of CAMHS. Unlike systematic reviews, SLRs allow researchers to start with broader questions, to explore the issue from different perspectives and perhaps find more comprehensive solutions that are not only effective, but also accounted for their feasibility and acceptability to key stakeholders. Unlike for systematic reviews, there is no strict requirement to rigorously adhere to SLR process and reviewers can skip or modify certain steps as they see fit. However, non-adherence to guidelines is likely to result in significant bias and compromise the reliability of findings. The framework we propose brings together numerous advancements made to SLR methodology, as well as introduces additional protocols to enhance methodological robustness, minimise bias, and ensure reliability of findings.

## Recommendations

 ▪ Assemble a research team with a range of skills and expertise.

▪ Draw on expertise of external partners, particularly practitioners, decision-makers and commissioners who will be translating findings into practice.

▪ Document in the review process in detail. Note rationale for each decision and consider how it would impact on generalisability and utility of findings.

▪ Use systematic procedures for literature searchers, selection of studies, data extraction and reporting. Keep detailed records to ensure replicability.

▪ If feasible, appraise the quality of included evidence.

▪ Be transparent about limitations of findings.

▪ Commit to and embrace open science principles.

## Supplementary information

**Additional file 1: Appendix 1.** Table 1: Search terms used in evidence charting searches. Table 2. Initial inclusion/exclusion criteria for evidence charting searches. Table 3: Results of evidence charting search. Table 4: Search terms used in final SLR searches.

**Additional file 2: Appendix 2.** Figure 1: CAMHS delivery models – logic map.

**Additional file 3: Appendix 3.** Executive summaries of findings for the exemplar review.

## Data Availability

All data and materials relating to the exemplar systematic review can be requested from Dr. Joanna Anderson.

## Abbreviations

SLR: Scoping literature review
CAMHS: Children and Adolescent Mental Health Services
MESH: Medical subject headings
CYP: Children and young people
PCC: Population, concept and context criteria
MH: Mental health
CBT: Cognitive-behavioural therapy
