# Supplementary material for: Advancing methodology for scoping reviews: recommendations arising from a scoping literature review (SLR) to inform transformation of Children and Adolescent Mental Health Services
Source: BMC Med Res Methodol. 2020 Sep 29;20:242. doi: 10.1186/s12874-020-01127-3 (PMC7526176; doi:10.1186/s12874-020-01127-3)
Supplement: Supplementary file 1 — Additional file 1: Appendix 1. Table 1: Search terms used in evidence charting searches. Table 2. Initial inclusion/exclusion criteria for evidence charting searches. Table 3: Results of evidence charting search. Table 4: Search terms used in final SLR searches. [file 12874_2020_1127_MOESM1_ESM.docx]

**Appendix 1**

**Table 1: Search terms used in evidence charting searches.**

| Mental health services AND | | | | |
| --- | --- | --- | --- | --- |
| AB ( (mental health N2 care) OR (mental N2 healthcare) OR (mental health N2 service*) OR (CAMHS) OR (mental health problem*) OR TI ( (mental health N2 care) OR (mental N2 healthcare) OR (mental health N2 service*) OR (CAMHS) OR (mental health problem*) | | | | |
| Children and adolescents AND | | | | |
| AB ( child* OR adolescen* OR teen* OR young people OR young person* OR minor* OR youth* OR infan*) OR TI ( child* OR adolescen* OR teen* OR young people OR young person* OR minor* OR youth* OR infan*) | | | | |
| Key priorities | | | | |
| Prevention and promotion:  AB (prevent* OR health promot* OR educat* OR inform* OR engage* OR teach* OR aware* ) OR TI ( prevent* OR health promot* OR educat* OR inform* OR engage* OR teach* OR aware* ) OR (MH "Primary Prevention+") OR (MH "Health Promotion+") | **Screening and identification:**  AB ( identif* OR screening OR detect* OR (early N3 (identification OR intervention)) OR initial assessment ) OR TI ( identif* OR screening OR detect* OR (early N3 (identification OR intervention)) OR initial assessment ) OR (MH "Early Intervention (Education)") | **Access to CAMHS:**  AB (access* OR referr* OR (open access) or (referral based) or (wait* time*) ) OR TI ( access* OR referr* OR (open access) or (referral based) or (wait* time*) ) OR (MH "Health Services Accessibility+") | **Provision of CAMHS:**  AB ((therapeutic alliance) OR (service* N2 (provision OR deliver* OR design*)) OR ((integrated OR coordinated OR co-located ) N2 service*) OR (continuity of care) OR liaison OR advocacy OR TI (therapeutic alliance) OR (service* N2 (provision OR deliver* OR design*)) OR ((integrated OR coordinated OR co-located ) N2 service*) OR (continuity of care) OR liaison OR advocacy | **Service evaluation and improvement:**  ((quality N3 (assess* OR indicator* OR improv* OR standard* OR assur* OR care)) OR ((user* OR patient*) AND feedback) OR TI ((quality N3 (assess* OR indicator* OR improv* OR standard* OR assur* OR care)) OR ((user* OR patient*) AND feedback) OR (MH "Quality of Health Care+") OR (MH "Quality Assurance, Health Care+") OR (MH "Quality Indicators, Health Care") |

**Table 2: Initial inclusion/exclusion criteria for evidence charting searches**

|  | Exclude if: |
| --- | --- |
| 1 | Not written in English. |
| 2 | Published before January 1990. |
| 3 | Not empirical, not evidence based, not reviews of other studies or not a policy document/guideline (exclude commentaries, letters, book reviews). |
| 4 | Not directly or indirectly focused on mental health service users age 0-25 years (i.e. studies with parents/carers of mental health service users, service providers will be included). |
| 5 | No focus on mental health or mental disorders. |
| 6 | Services are not delivered in community settings |
| 7 | Describes children and adolescents mental health services in developing countries (according to World Economic Situation and Prospects 2015). |

**Table 3: Results of evidence charting search**

| Area of CAMHS provision | Total abstracts retrieved | Of 2000 most relevant abstracts: | | Specific areas identified |
| --- | --- | --- | --- | --- |
|  |  | **Abstracts included** | **Abstracts excluded** |  |
| Prevention and promotion of MH and wellbeing | 11399 | 105 | 1895 | Community based prevention  School based prevention  Suicide prevention  Substance abuse prevention  Education and rising awareness  Web-based interventions  Literature reviews  Policy and guidelines |
| Identification | 7628 | 123 | 1877 | Early intervention  Screening tools  School based screening  Screening in healthcare settings  Literature reviews  Policy and guidelines |
| Access to CAMHS | 5103 | 118 | 1822 | Access/referrals (general)  Improving access  Barriers for access/referral  Users’ experience  Waiting times  Pathways  Literature reviews |
| Provision of CAMHS | 1495 | 160 | 1885 | Service delivery models  Integrated/comprehensive services  Development/redesign/implementation  Service evaluation  Advocacy  Therapeutic alliance |
| Service evaluation and improvement | 1495 | 160 | 1335 | Quality indicators/service evaluation  Quality improvement initiatives  Users’ experience/satisfaction  Outcome monitoring |

**Table 4: Search terms used in final SLR searches**

| Mental health services AND | | | | | |
| --- | --- | --- | --- | --- | --- |
| AB ( (mental health N2 care) OR (mental N2 healthcare) OR (mental health N2 service*) OR (CAMHS) OR (mental health problem*) OR (mental* N3 (health OR ill*)) OR TI ( (mental health N2 care) OR (mental N2 healthcare) OR (mental health N2 service*) OR (CAMHS) OR (mental health problem*) OR (mental* N3 (health OR ill*)) | | | | | |
| Children and adolescents AND | | | | | |
| AB ( child* OR adolescen* OR teen* OR young people OR young person* OR minor* OR youth* OR infan*) OR TI ( child* OR adolescen* OR teen* OR young people OR young person* OR minor* OR youth* OR infan*) | | | | | |
| Prevention and promotion | | | | | |
| School based prevention: AB ( (classroom-based N3 (prevent* OR promot*)) OR ((prevent* OR promot*) N3 school*) ) OR TI ( (classroom-based N3 (prevent* OR promot*)) OR ((prevent* OR promot*) N3 school*) ) | | **Education and rising awareness:** AB (educat* OR aware* OR attitude* OR literacy OR inform*) OR TI (educat* OR aware* OR attitude* OR literacy OR inform*) | | **Suicide prevention**: AB (“suicide prevention” N3 programme) OR suicidal OR TI (“suicide prevention” N3 programme) OR suicidal | |
| Screening and identification | | | | | |
| School based screening: AB (school* N2 (screening OR identif* OR detect* OR assess*)) OR TI (school* N2 (screening OR identif* OR detect* OR assess*)) | | **Screening in healthcare settings**: AB ( paediatric* OR pediatric* OR (primary care) OR GP OR (general practi*) OR (family practi*) OR (emergency department) OR A&E ) OR TI ( paediatric* OR pediatric* OR (primary care) OR GP OR (general practi*) OR (family practi*) OR (emergency department) OR A&E ) | | **Screening tools:** AB ( child* OR adolescen* OR teen* OR young people OR young person* OR minor* OR youth* OR infan* ) OR TI ( child* OR adolescen* OR teen* OR young people OR young person* OR minor* OR youth* OR infan* ) | |
| Access to CAMHS | | | | | |
| Barriers for access and referral: AB ( (barrier* OR problem* OR delay*) N3 (access* OR referr* OR engage* OR utili*) ) OR TI ( (barrier* OR problem* OR delay*) N3 (access* OR referr* OR engage* OR utili*) ) | | **Wait times:** AB ( (wait* N3 (time* OR list)) OR (access time*) ) OR TI ( (wait* N3 (time* OR list)) OR (access time*)) | | **Improving access:** AB ( ((improv* OR enhanc*) N3 (access* OR refer* OR engag*)) ) OR TI ( ((improv* OR enhanc*) N3 (access* OR refer* OR engag*)) ) | |
| Provision of CAMHS | | | | | |
| Service delivery models: AB ( model* OR system* OR deliver* ) OR TI ( model* OR system* or deliver* ) | | **Integrated/comprehensive services:** AB ( (integrate* OR coordinate* OR co-locate* OR comprehensive) ) OR TI ( (integrate* OR coordinate* OR co-locate* OR comprehensive) ) | | **Technology enabled MH interventions:** AB (((web based OR web-based OR online OR website* OR internet OR internet-based OR mobile OR phone* OR smartphone* OR computer*) N4 (intervention* OR programme* OR program* OR application* OR app* OR app-based OR technolog*)) OR e-health OR m-health) OR TI (((web based OR web-based OR online OR website* OR internet OR internet-based OR mobile OR phone* OR smartphone* OR computer*) N4 (intervention* OR programme* OR program* OR application* OR app* OR app-based OR technolog*)) OR e-health OR m-health) | |
| Service evaluation and improvement | | | | | |
| Service redesign and implementation: AB ( (service N3 (design* OR redesign* or re-design* OR chang*)) OR (implement* N3 (process* OR framework* OR theor* OR model*)) OR (chang* AND process*) ) OR TI ( (service N3 (design* OR redesign* or re-design* OR chang*)) OR (implement* N3 (process* OR framework* OR theor* OR model*)) OR (chang* AND process*) ) | **Quality indicators:** AB ( (quality AND (assess* OR indicator* OR standard* OR assur* OR improv* OR measur*)) ) OR TI ( (quality AND (assess* OR indicator* OR standard* OR assur* OR improv* OR measur*)) ) | | **Outcome monitoring:** AB (( outcome* AND measure* OR monitor* OR domain*) OR (self-report AND measure* OR outcome*)) OR TI (( outcome* AND measure* OR monitor* OR domain*) OR (self-report AND measure* OR outcome*)) | | **Users experience/satisfaction:** AB ( ((user* OR patient* OR parent* OR carer*) AND (experienc* OR feedback OR satisfy*OR opinion* OR perc* OR view* OR perspective*)) ) OR TI ( ((user* OR patient* OR parent* OR carer*) AND (experienc* OR feedback OR satisfy*OR opinion* OR perc* OR view* OR perspective*)) ) |
