# Supplementary figures and images for: Advancing methodology for scoping reviews: recommendations arising from a scoping literature review (SLR) to inform transformation of Children and Adolescent Mental Health Services
Source: BMC Med Res Methodol. 2020 Sep 29;20:242. doi: 10.1186/s12874-020-01127-3 (PMC7526176; doi:10.1186/s12874-020-01127-3)

Appendix 3: Logic map

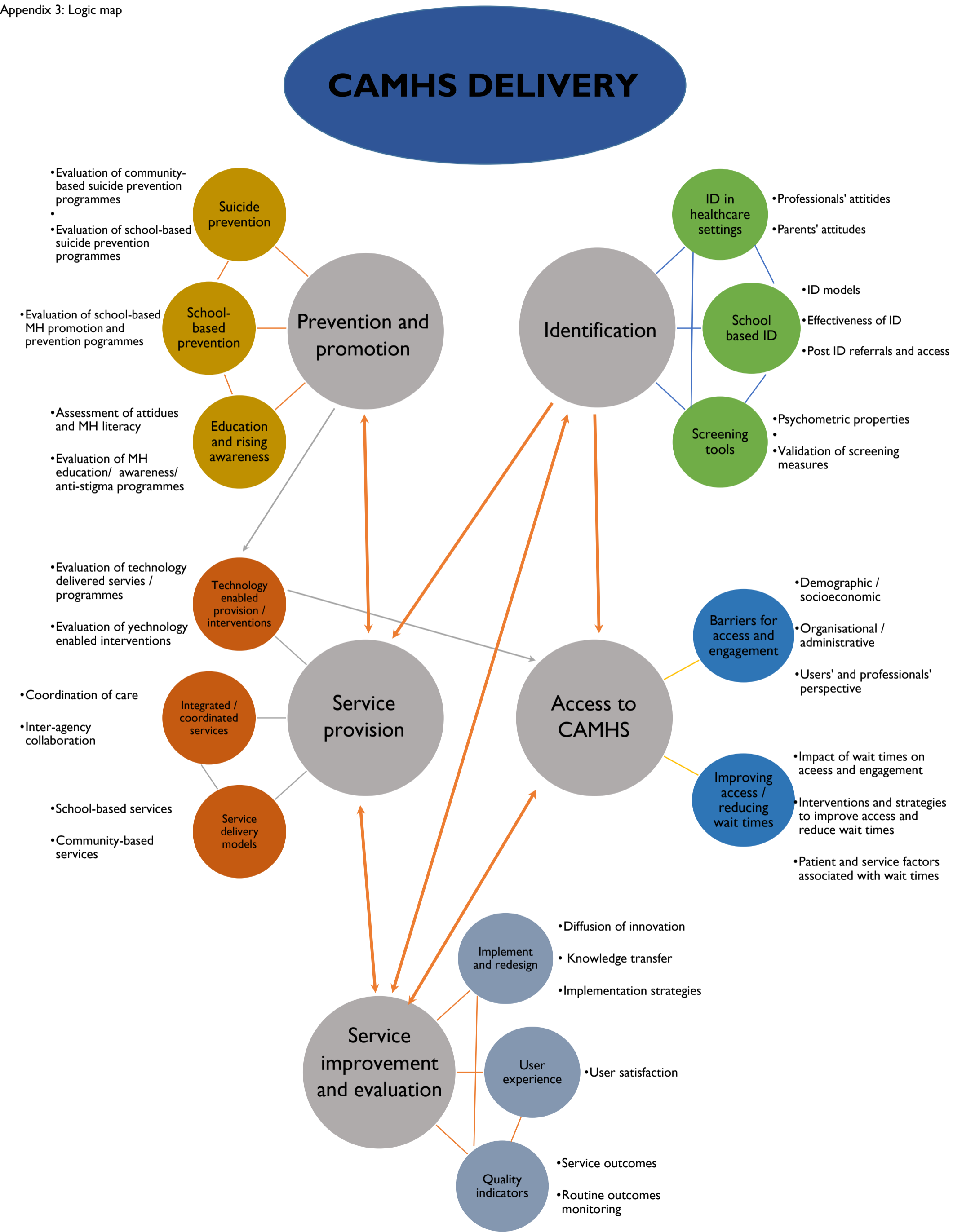

Supplement: Supplementary file 2 — Additional file 2: Appendix 2. Figure 1: CAMHS delivery models – logic map. [file 12874_2020_1127_MOESM2_ESM.pdf]
