# Supplementary material for: Advancing methodology for scoping reviews: recommendations arising from a scoping literature review (SLR) to inform transformation of Children and Adolescent Mental Health Services
Source: BMC Med Res Methodol. 2020 Sep 29;20:242. doi: 10.1186/s12874-020-01127-3 (PMC7526176; doi:10.1186/s12874-020-01127-3)
Supplement: Supplementary file 3 — Additional file 3: Appendix 3. Executive summaries of findings for the exemplar review. [file 12874_2020_1127_MOESM3_ESM.pdf]

Appendix 2

What prevents children and young people with mental health difficulties from accessing and engaging with Children and Adolescent Mental Health Services?

The NIHR Applied Research Collaborative East of England consulted Children and Adolescent Mental Health Services' (CAMHS) users and professionals working with children and young people with mental health difficulties how CAMHS could better address the needs of young people. One of the key messages was the need to improve access and engagement with CAMHS, so we collated and analysed research evidence about the barriers to access for CYP and their families, as well as how best to address them.

Why is this important?

Delay in addressing children and young people's mental health difficulties can have long-term, negative impact on individuals' functioning, health and wellbeing (Joint Commissioning Panel for Mental Health, 2013). Children and young people with poor mental health have lower grades, are more likely to be excluded or drop-out of school, and are at higher risk of substance abuse, delinquency, self-harm and suicide (Patel et al. 2007). Most adults with poor mental health first experience difficulties before their mid-teens (Kim-Cohen et al. 2003).

In the United Kingdom, one in eight (12.8%) children and young people aged 5 to 19 year olds has at least one mental disorder (Sadler et al, 2018). Yet, only 25% of those with a disorder reach CAMHS (Mandalia et al., 2018), while service-level barriers are one of the key factors in delaying children and young people's access to CAMHS (Anderson et al, 2017).

Our findings: key factors to consider when planning CAMHS services

Information

- Is there transparent, up-to-date information about available services and how and how to access?
- Is this easily accessible for young people, parents, schools and GPs?

Flexibility

- Is a service offers appointments outside of children's school and parent's working hours?
- Are services/appointments available at convenient locations?

Procedures

- Are referral procedures straighforward?
- Do agencies communicate with each other if a child/young person is seeing multiple specialists?

Providers

- Do CAMHS clinicans:
  - Listen to children, young people and their and families' concerns?
  - Involve chilren, young people and their families in making decisions about treatment and support?
  - Respect confidentiality?

Headline findings

CYP and families identified the following barrier for access and engagement with CAMHS:

Availability:

- Lack of availability of CAMHS services especially for CYP and families living in rural and remote areas. Travelling significant distances is problematic for CYP and parents, especially if they do not have an access to a car, and public transport is scarce or unreliable.
- Lack of culturally appropriate CAMHS services and language barriers prevents CYP and families from racial and ethnic minority groups from accessing support.

Poor information:

- Not knowing where services are located, how to obtain a referral and make an appointment are major barriers for access for young people, especially if they do not want to involve their parents or school.
- Many families find out about available support through informal routes e.g. social media groups or from other parents whose children are experiencing similar difficulties.

Inflexibility:

- Parents find it difficult to repeatedly take time off work, and take the child out of school, if their child's treatment is ongoing.
- Lack of communication between services and providers within the same service results in treatment delays and disruptions.

Procedures:

- Parents find it hard to understand and navigate complex, multi-step referral processes, which often deters them from pursuing mental health support.
- Parents report failing to access support for their child because they did not push hard enough. Personal style, lack of education and lack of time resulting from work obligations and parenting of other children were problematic for those who struggled to gain access.

Wait times:

- GPs and school staff reported 'not seeing the point' of referring CYP to CAMHS due to very long waiting list, and the high likelihood that a referral will not be accepted.
- CYP and families, when facing a long wait, often disengage and give up

Providers' attitudes:

- Parents often report having difficulties convincing a provider that their child has a problem. Dismissive responses frequently discouraged parents from further help-seeking.
- Parents often felt blamed for their child's difficulties.
- Both parents and CYP thought they were not provided with enough information
- Some reported not understanding or being excluded from making decisions about treatment.
- CYP reported concerns about privacy and confidentiality.

For more information see: Anderson et al. (2017) A scoping literature review of service-level barriers for access and engagement with mental health services for children and young people.

How can we improve children and young people access and engagement with Children and Adolescent Mental Health Services?

The NIHR Applied Research Collaborative East of England consulted Children and Adolescent Mental Health Services' (CAMHS) users and professionals working with children and young people with mental health difficulties how CAMHS could better address the needs of young people. One of the key messages was the need to improve access and engagement with CAMHS, so we collated and analysed research evidence about this.

Why is this important?

In the United Kingdom, one in eight (12.8%) children and young people aged 5 to 19 year olds has at least one mental disorder (Sadler et al, 2018). Yet, only 25% of those with a disorder reach CAMHS (Mandalia et al., 2018). Delay in addressing children and young people's mental health difficulties can have long-lasting negative consequences for individuals, their families (Patel et al. 2007) and societies (Joint Commissioning Panel for Mental Health, 2013).

Many studies point out at long waiting times and inflexible, inaccessible services as key factors deterring children, young people and their families from seeking help and engaging with CAMHS (Anderson et al, 2017). We systematically studied factors that might make it easier for CYP and their families to access CAMHS and engage with interventions.

Our findings: strategies to improve access and engagement with CAMHS

Location

- Provide services in easily accessible locations like school, GP surgeries, community centres.
- Provide outreach services for children and young people living in rural and remote locations.

Flexibility

- Offer appointments outside of children's school and parents' working hours.
- Consider establishing walk-in and self-referral clinics.

Prioritisation

- Dedicate time for evaluation appointments, and prioritise access based on severity.
- Use standardised tools to help case assessment and prioritisation.

Communication

- Contact families to:
  - Provide updates on how much longer they will wait for their appointment.
  - Confirm they want to continue waiting for services.

Headline findings

The following strategies increase access and engagement with CAMHS:

Providing services in accessible locations:

- Providing services in schools, local GP surgeries and community centres improve access and engagement, especially for users from minority and hard to reach groups.
- Providing services in schools improves access for young people who would have not sought help through traditional referral pathways.
- Establishing outreach clinics improves access for young people living in rural and remote locations.

Facilitated access:

- Availability of walk-in clinics improves access for young people who are otherwise discouraged by complicated referral procedures.
- Although walk-in clinics users generally present with more severe mental health difficulties compared to usual care, they improve more quickly and require fewer appointments.
- Self-referral services are more acceptable for young people and families from ethnic minority groups.

Prioritisation:

- Dedicating time for evaluation appointments and case allocation based on outcomes of these initial consultations reduces wait times and 'did not attend' rate.
- Using standardised tools to facilitate prioritisation helps reducing wait times. Example of tools used: Highland Prioritisation Criteria, The Western Canada Waiting List Project tool, Child Mental Health Priority Criteria Score (CMH-PSC), The Finish Child Psychiatric Criteria Tool

Communication:

- Regular communication with families about remaining wait time, and asking parents to confirm that they want to remain on the waiting list can help reduce 'did not attend rates'.
- Sending questionnaires for children and young people to filled in and bring to their first appointment significantly reduces non-attendance.

How schools can effectively identify children and young people with mental health difficulties?

Schools are an ideal setting for early identification of mental health difficulties (Humphrey, Wigelsworth, 2016) due to nearly universal access to children and young people, including those from marginalised population (Green et al, 2004) and large number of contact hours (Eklund et al, 2014). The green paper published in 2017 sets out UK Government's plan for schools to assume a leading role in the early identification of children and young people experiencing mental health difficulties (Department of Health and Social Care & Department for Education, 2017). Early identification with the provision of support in schools may improve access to care for children and young people with complex mental health needs.

Why is this important?

In the United Kingdom, one in eight (12.8%) children or young people aged 5 to 19 years old has at least one mental disorder (Sadler et al, 2018), yet only 25% access mental health services (Mandalia et al., 2018). Delay in addressing children and young people's mental health difficulties can have a long-term, negative impact on individuals' functioning, health and wellbeing (Joint Commissioning Panel for Mental Health, 2013). Mental health difficulties in children and young people, unless treated, tend to persist (Ford et al, 2017) while three quarters of those with mental health conditions in early adulthood experience difficulties prior to the age 18 (Kim-Cohen et al. 2003). We collated and analysed evidence about identification of mental health difficulties in schools, and what is the most effective way of doing it.

Our findings: How schools can effectively identify pupils with mental health difficulties

Four Identification methods

- **Universal screening**, compared to other methods identifies more children, including those with internalising disorders. Using verified, reliable screening questionnairs helps ensure accurate results.

- **School staff nomination** often overlooks children with internalising disorders. Accuracy of staff nomination can be improved with relevant training.

- Methods relying solely on **standard information collected by schools** (e.g. grades, detention/truancy/absence records) are the least accurate.

- Using a **combination** of the above methods improves the accuracy of identification.

Implementation

- Succesfully adopted identifiction programmes are:

  - **Flexible:** schools can tailor them to fit their unique context and requirements.

  - **Rapid**: require minimum input from school staff and are not intrusive into student time

  - **Straighforward:** have a minimal number of steps and tasks to be completed by school staff and students

  - **Low cost**: ideally do not require additional resources beyond those already available in schools
